# Circadian rhythm dysfunction in bipolar affective disorder

**DOI:** 10.1192/j.eurpsy.2021.521

**Published:** 2021-08-13

**Authors:** M.T. Valadas, R. Mota Freitas

**Affiliations:** 1 Serviço De Psiquiatria, Unidade Local de Saúde do Baixo Alentejo, Beja, Portugal; 2 Departamento De Psiquiatria E Saúde Mental, Hospital do Espírito Santo de Évora, Évora, Portugal

**Keywords:** bipolar affective disorder, Circadian rhythm

## Abstract

**Introduction:**

Sleep is paramount in bipolar affective disorder and sleep disturbance can be a trigger or initial manifestation of an episode of illness. Changes in the circadian rhythm in bipolar affective disorder have consistently been recognized and reported, however, this feature can be overlooked in daily clinical practice.

**Objectives:**

We aim to review and summarize the literature regarding changes in circadian rhythm in patients with bipolar affective disorder.

**Methods:**

We performed an updated review in the PubMed database using the terms “circadian rhythm” and “bipolar affective disorder”.

**Results:**

Irregularity of the sleep–wake rhythm, eveningness chronotype, abnormality of melatonin secretion, vulnerability of clock genes, and the irregularity of social time cues are circadian rhythm markers disrupted in bipolar affective disorder. Circadian rhythm dysfunction might be a trait marker of this illness and can act as a predictor for the first onset of bipolar affective disorder and the relapse of mood episodes. Achieving normalization of circadian rhythm in combination with pharmacological, psychosocial and chronobiological treatments can be a tool for managing bipolar affective disorder.

**Conclusions:**

Recognizing patterns of changes in circadian rhythms is important to detect and diagnose bipolar disorder in clinical practice, also affecting treatment. These alterations are often overlooked and can lead to inadequate treatment and management.

